# Validation of eyes-closed resting alpha amplitude predicting neurofeedback learning of upregulation alpha activity

**DOI:** 10.1038/s41598-021-99235-7

**Published:** 2021-10-04

**Authors:** Ken-Hsien Su, Jen-Jui Hsueh, Tainsong Chen, Fu-Zen Shaw

**Affiliations:** 1 Department of Biomedical Engineering, National Cheng Kung University, Tainan, Taiwan; 2 Mind Research and Imaging Center, National Cheng Kung University, Tainan, Taiwan; 3 Department of Psychology, National Cheng Kung University, No. 1 University Road, Tainan, 70101 Taiwan

**Keywords:** Electroencephalography - EEG, Functional clustering

## Abstract

Neurofeedback training (NFT) enables users to learn self-control of EEG activity of interest and then to create many benefits on cognitive function. A considerable number of nonresponders who fail to achieve successful NFT have often been reported in the within-session prediction. This study aimed to investigate successful EEG NFT of upregulation alpha activity in terms of trainability, independence, and between-session predictability validation. Forty-six participants completed 12 training sessions. Spectrotemporal analysis revealed the upregulation success on brain activity of 8–12 Hz exclusively to demonstrate trainability and independence of alpha NFT. Three learning indices of between-session changes exhibited significant correlations with eyes-closed resting state (ECRS) alpha amplitude before the training exclusively. Through a stepwise linear discriminant analysis, the prediction model of ECRS’s alpha frequency band amplitude exhibited the best accuracy (89.1%) validation regarding the learning index of increased alpha amplitude on average. This study performed a systematic analysis on NFT success, the performance of the 3 between-session learning indices, and the validation of ECRS alpha activity for responder prediction. The findings would assist researchers in obtaining insight into the training efficacy of individuals and then attempting to adapt an efficient strategy in NFT success.

## Introduction

Neurofeedback training (NFT) enables users to learn self-regulation of their cortical oscillations by receiving moment-to-moment feedback from their electroencephalogram (EEG)^1,2^. NFT is a safe, inexpensive, and accessible technology that is a valuable intervention. Several lines of evidence have demonstrated NFT as a promising and nonpharmacological supportive treatment for neurological and psychiatric disorders, such as attention deficit hyperactivity disorder (ADHD)^3,4^, depression^5^, anxiety^6^, or insomnia^7^. NFT has also been applied in healthy participants to enhance several aspects of cognitive functions^8–11^. These studies have indicated that a well-trained performance is very important in either cognitive enhancement or symptom amelioration.

Ideally, participants can learn to control their brain activities through NFT assistance. Although many participants can gain successful EEG learning by a variety of NFT protocols, some participants fail to achieve the required control of brain activity in a desired direction^9,12^. Of these studies, participants were classified into the category of responders or nonresponders. The rate of responders varies in a range of 50–80%^9,11,13^. Responders express a significant improvement in performance after training in a variety of NFT protocols^8,14–17^. In contrast, nonresponders often show less improvement in behavioral outcomes than responders^18,19^ or even no improvement after NFT^20^. Thus, responder identification may play an important role in the efficacy of NFT.

Regarding responder identification, numerous studies focus on the learning ability of an NFT. Previous NFT studies have shown controversial results in training performance and outcomes^21,22^. A previous study proposed successful training for responders in terms of trainability (amplitude change throughout the NFT) and independence (no alteration out of trained frequency bands)^9^. In addition to these two characteristics, an important contribution to training success is prediction from psychological or neurophysiological variables (for review^23,24^). The prediction of successful NFT would have great advantages in reducing potential frustration, saving cost on nonresponders, modifying the training protocol, and further understanding the clue of poor learning ability on NFT.

The neurophysiological features, particularly initial brain activity before training, have been investigated to predict NFT success. For instance, learning beta/theta control can be predicted by resting beta activity prior to training^25^. Additionally, learning of the sensorimotor rhythm or alpha rhythm can be predicted by the amplitude/power of initial sensorimotor rhythm^26^ or alpha activity^27^ combined with other frequency bands, respectively. EEG NFT learning is evaluated by the training parameter change within sessions^26,28,29^, across sessions^25,27^, within sessions compared to baselines^30^, or across sessions compared to baselines^11,31^. The within-session performance indicates changes of a testing day but not the overall progression for an NFT. Responders of an NFT are typically defined by significant changes across sessions, e.g., between the first session and the last session^9,32^ or between the first session and other sessions^2^. This is a cross-day evaluation. In contrast to those prediction studies using within-session assessment, there is largely a lack of clarity regarding the performance of between-session alteration by the predictor from initial amplitude of brain activity^2^.

Several aspects of between-session learning indices have been investigated to evaluate trained performance regarding initial amplitude^24^. One of the most popular learning indices is the alteration of EEG amplitude or power between the first session and the last session^9,32^. A previous study demonstrated the power of sensorimotor rhythm in the middle process of training, which is calculated as the average difference between the power of the first session and the other sessions, as a great feature^2^. Another between-session learning index is the regression level from changes in alpha amplitude throughout all sessions, which exhibits a remarkable linear relation with initial alpha amplitude^27^. Although these learning indices exhibit strong correlations with the initial amplitude of trained rhythm individually^2,27^, there is no systematic comparison of these learning indices for prediction with the initial EEG amplitude of interest.

Stepwise linear discriminant analysis (LDA) combined with leave-one-out cross-validation (LOOCV) is commonly used for responder validation regarding the learning index and initial EEG amplitude. An inconsistent phenomenon between the trained brain activity and validated model of brain activity has been exhibited in previous studies^25,26,29^. A previous study of beta/theta ratio NFT used a 2-parameter model (i.e., sigma power of baseline and beta1 power of the first training block) to validate the responder of a learning index^25^. The upregulation of sensorimotor NFT used a topographic model of sensorimotor amplitudes from 40 channels to validate responders regarding the within-session learning index^26^. The downregulation alpha NFT used a 4-parameter model from amplitudes of theta, lower alpha, sigma, and beta1 to validate responders from a within-session learning index^29^. The features and learning indices used in the validation model are very divergent among these studies. In addition, NFT success can be predicted by the relative alpha amplitude prior to an NFT of upregulation alpha activity through regression analysis of the within-session and between-session learning indices^27^. However, the relation between the learning indices and amplitudes of other frequency bands is unknown in the upregulation alpha NFT. Moreover, the predictability of the upregulation of alpha NFT by learning indices has not yet been validated.

The present study carried out NFT to upregulate alpha activity because previous studies have demonstrated advantages of alpha NFT in memory enhancement^9,11,31,33^. We anticipated trainability and independence of alpha activity in the NFT process. The present study calculated 3 between-session learning indices and assessed their correlations with eyes-closed resting state (ECRS) EEGs before training. Furthermore, we validated the prediction of ECRS EEGs on the 3 between-session learning indices through stepwise LDA with LOOCV. We hypothesized a successful alpha NFT with good trainability and independence and predictability of NFT success from prior ECRS brain activity.

## Results

### Trainability and independence of NFT

**Figure 1:**
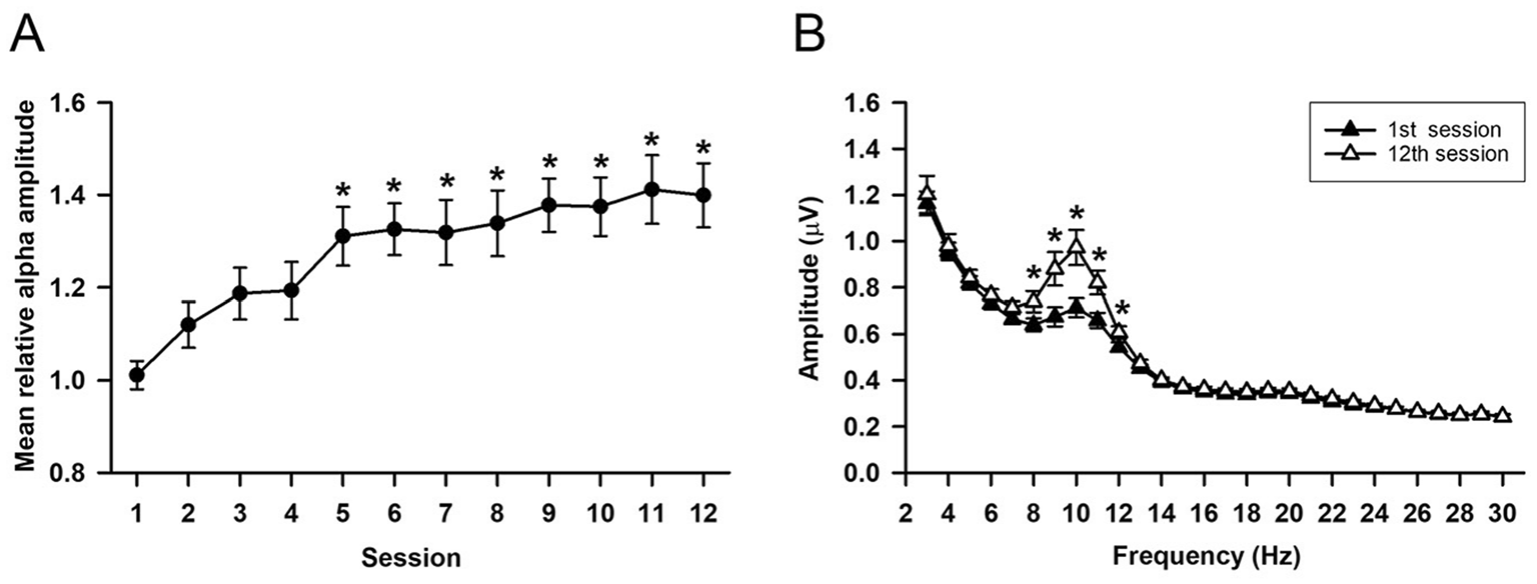
Neurofeedback training (NFT) of upregulation alpha activity. (**A**) The mean relative alpha amplitude (MRAA) throughout 12 NFT sessions. (**B**) Amplitude of 3–30 Hz between the 1st and 12th sessions. The error bars represent the standard error of the mean (SEM). *P < 0.05 vs. 1st session.

Statistical analysis of baseline alpha amplitude revealed no significant main effect of session (F_11,495_ = 1.53, P = 0.12) and no significant difference between the first session and other sessions. Figure 1 shows the spectrotemporal progression of EEG throughout the entire NFT. Obviously, the mean relative alpha amplitude (MRAA) showed progressive elevation as the training session increased (Fig. 1A). The MRAA exhibited a significant main effect on the session factor (F_11,495_ = 9.49, P < 0.05). The MRAAs from the 5th to 12th sessions were significantly higher than those of the first session. Moreover, the amplitude spectra of the first and 12th sessions were remarkably different in the alpha frequency band (AFB) exclusively (Fig. 1B). There was a significant main effect of session (F_1,1215_ = 8.52, P < 0.05). In particular, amplitudes of the 12th session exhibited significant increments in the range of 8–12 Hz compared with those of the first session. Thus, our results exclusively indicated successful regulation over EEGs of 8–12 Hz.

### Relation between ECRS alpha amplitude and learning indices

**Figure 2:**
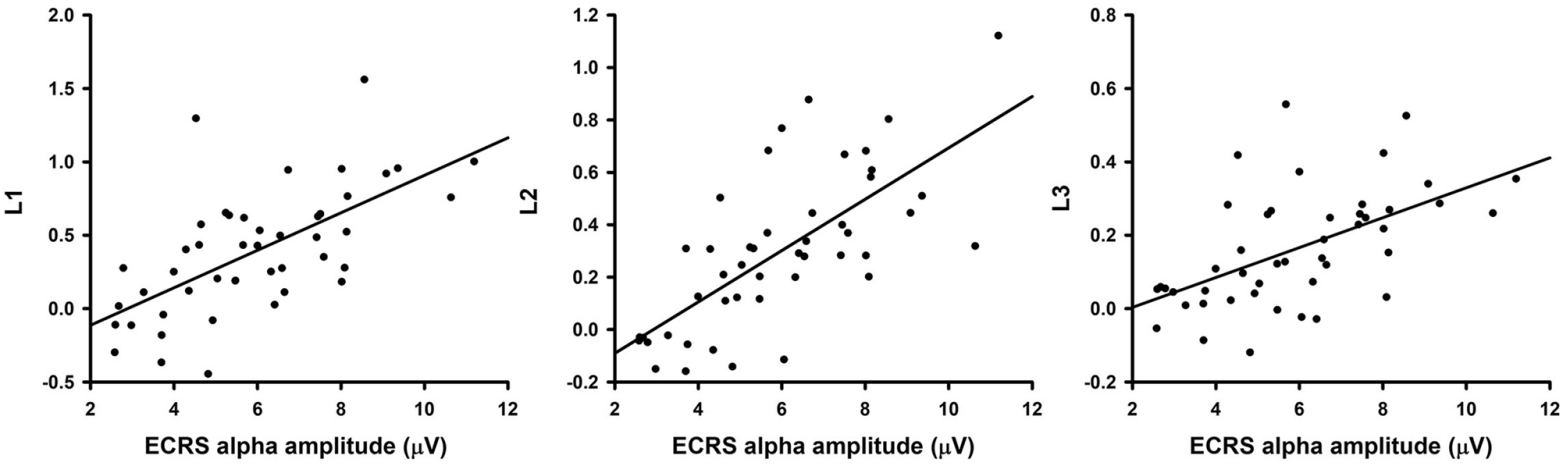
Correlation of the eyes-closed resting-state (ECRS) alpha amplitude with 3 learning indices (L1, L2, and L3 from left to right). Each dot corresponds to one subject.

All learning indices were derived from the progression of alpha activity during NFT in this study. The learning index L1 ranged from − 0.45 to 1.56 (mean ± SD: 0.39 ± 0.43), L2 ranged from − 0.16 to 1.12 (0.29 ± 0.30), and L3 ranged from − 0.12 to 0.56 (0.16 ± 0.16). We evaluated the correlation between the learning indices and ECRS EEGs prior to the training and found that the ECRS alpha amplitude exhibited significant positive correlations with the learning indices of L1 (r = 0.64, P < 0.001), L2 (r = 0.70, P < 0.001), and L3 (r = 0.55 P < 0.001) (Fig. 2). In contrast, delta, theta, and beta amplitudes of ECRS EEGs showed no significant correlation with the three learning indices (Table 1). This study further selected ECRS alpha amplitude as the predictor of learning indices, and then the linear regression model R^2^ was 0.410 for L1 (P < 0.001), 0.492 for L2 (P < 0.001), and 0.30 for L3 (P < 0.001). Therefore, the ECRS alpha amplitude was identified as a significant predictor that accounted for 41.0% of the variance in L1, 49.2% of the variance in L2, and 30% of the variance in L3. Accordingly, the ECRS alpha amplitude provided the best prediction in L2.

**Table 1:** Pearson correlations between 4 characteristic frequency bands of ECRS EEG and learning indices.

	L1	L2	L3
Delta	r = 0.25	r = 0.12	r = 0.13
Theta	r = 0.32	r = 0.14	r = 0.16
Alpha	r = 0.64*	r = 0.70*	r = 0.55*
Beta	r = 0.13	r = − 0.02	r = − 0.04

*P < 0.05.

### Classification of responders and nonresponders

As the learning objective was to increase alpha activity during an NFT, the participant with a positive learning index was defined as the responder and vice versa. The numbers of responders for L1, L2, and L3 were 38 (82.6%), 35 (76.1%), and 41 (87.0%), respectively. Furthermore, stepwise LDA was applied to extract valuable features and build a prediction model according to ECRS EEGs for responder classification. First, this study examined the amplitudes of 4 typical frequency bands (i.e., delta, theta, alpha, and beta) of ECRS EEGs. Alpha amplitude was the only significant parameter for responder classification on all learning indices. Then, the amplitude of AFB in ECRS EEGs was extracted to build the prediction model.

**Figure 3:**
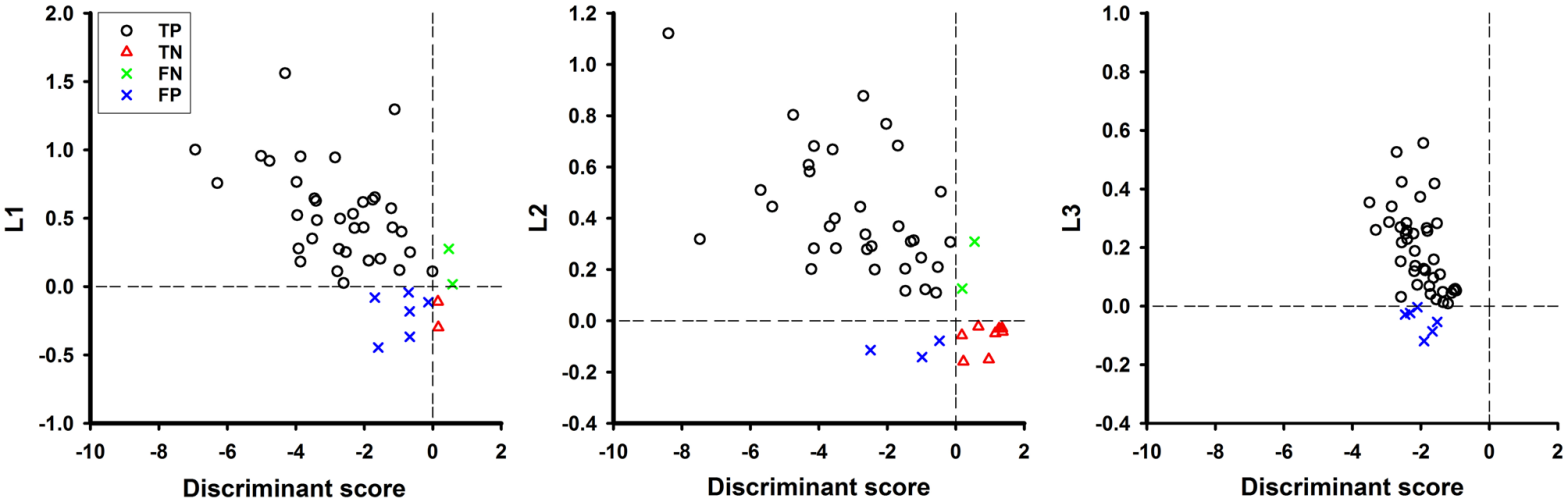
Cross-validation for ECRS discriminant score of the prediction model from ECRS’s alpha amplitude regarding 3 learning indices (L1, L2, and L3). The vertical dashed line indicates the boundary for responder classification from the discrimination score of an ECRS alpha amplitude model. The horizontal dashed line indicates the boundary for responder classification from a learning index. Each symbol represents a participant as either responder with right prediction (TP, black circle), nonresponder with right prediction (TN, red triangle), responder with wrong prediction (FN, green cross), or nonresponder with wrong prediction (FP, blue cross).

Moreover, we further validated the prediction ability of the AFB model on responder identification regarding the three learning indices through LOOCV. A participant with a negative discriminant score of the AFB model was considered a responder and vice versa. We found a large portion of participants who were responders through cross-validation between the learning indices and discriminant score of the prediction model (Fig. 3). Table 2 summarizes the cross-validation results from the 3 learning indices against the discriminant score of the AFB model in terms of accuracy, sensitivity and specificity. The cross-validation accuracy of the AFB model was > 82% regarding all learning indices. The L1 index regarding the AFB model exhibited the lowest accuracy (82.6%) and moderate performance in sensitivity and specificity compared with the other two learning indices. The L3 index exhibited the highest sensitivity compared to the other two indices, but its specificity was zero. The specificity of the L1 and L3 indices was very low (< 30%). The L2 index exhibited the highest accuracy (89.1%) with high sensitivity and specificity (> 70%). The overall performance of the AFB model regarding the L2 index was best compared to the other conditions.

**Table 2:** Cross-validation performance between 3 learning indices and the prediction model of ECRS’s alpha frequency band.

	L1 (%)	L2 (%)	L3 (%)
Sensitivity	94.7	94.3	100.0
Specificity	25.0	72.7	0.0
Accuracy	82.6	89.1	87.0

## Discussion

Considering the importance of alpha specification on outcome measures and predictions, we aimed to investigate whether ECRS alpha activity prior to NFT exclusively predicts learning ability in successful NFT progression. This study achieved a successful NFT, presenting spectral changes of interest frequency band (independence) and temporal progression (trainability) throughout 12 training sessions. Only initial ECRS alpha activity was positively correlated with 3 learning indices that were derived from temporal alteration of alpha activity throughout the training course. Stepwise LDA exhibited alpha activity of ECRS EEG being a significant feature exclusively to discriminate responder from participants for all learning indices. Moreover, the prediction accuracy of the AFB model was high (> 82% in accuracy) in the leave-one-out cross-validation, particularly for the responder classification using the L2 index. The results suggest that a success of upregulation alpha NFT needs to exhibit an independent alpha change and that prior ECRS alpha activity predicts training success.

NFT success can be predicted by relative alpha amplitude prior to an NFT of upregulation alpha activity through regression analysis of the within-session and between-session learning indices^27^. The current study also found similar regression results. Moreover, this study provided additional evidence of the cross-validation on predictability for the 3 between-session learning indices. The best correlation with the ECRS alpha amplitude is the L3 learning index in a previous study^27^. However, the L3 learning index was worse than the other two indices in terms of the correlation coefficient and regression power in the present study. Possible reasons for the discrepancy between these two studies of the upregulation alpha NFT are different training sessions (12 vs. 20), trial amount of a session (6 vs. 12), and trial duration (6 min vs. 20 s). A meta-analysis study indicated that a long trial duration is good to increase alpha amplitude and duration during NFT^33^. The MRAA of the previous study ranged from 0.9 to 1.05 throughout 20 sessions^27^. MRAA throughout the training increases slowly with a linear trend. However, the MRAA of the present study ranged from 1 to 1.35 throughout 12 sessions. The increasing trend looks like a sigmoid curve rather than a linear trend, which may reduce the predictability of the ECRS alpha amplitude with the L3 index.

Responders to NFT have been reported to account for 50–80% of NFTs in previous studies according to different evaluations^9,11,13^. These previous studies use the performance of outcomes between the first and last sessions or the last 3 sessions as a statistical comparison to assess responders, which is similar to the learning index L1 here. The current study exhibited a high responder ratio (82.6%) for the L1 learning index, which reflects a large increase in MRAA during NFT. The high responder rate may contribute to high prediction and cross-validation accuracy by ECRS alpha activity.

A successful NFT has been proposed to use trainability and independence of training outcomes^9^, e.g., changes in amplitude and/or duration of trained brain activity. This study provided evidence of temporal amplitude changes in alpha activity throughout 12 sessions and significant alterations in the alpha frequency range exclusively to support trainability and independence for NFT. Recently, a previous study proposed 3 learning indices to investigate predictions of between-session, within-session, and across-all-sessions learning in an NFT^27^. Additionally, the same group has used a within-session learning index to validate the predictability of NFT responders using a 4-frequency-band model^29^. We also validated the predicted accuracy as 82.6–89.1% using the AFB model with 3 learning indices. The prediction of successful NFT would have great advantages in reducing potential frustration, saving costs on nonresponders, etc. In addition to trainability and independence, predictability for NFT participants can be considered performance parameters to motivate the learning driving force from participants for trainers in an NFT.

From a practical viewpoint, this study provided direct and economic advantages for trainers in the prediction of participant training through convenient and time-saving ECRS EEG recordings before training. In general, NFT usually needs tens or hundreds of sessions for patients with disorders, such as attention-deficit-hyperactivity-deficit symptoms^4,34^. Reducing training sessions and attaining effective advantages are always concerns and appreciations for NFT. The prediction protocol using the participant’s ECRS activity can help the researcher understand the potential characteristics of each participant and strengthen valuable instructions to increase the training progress of an NFT. For instance, ECRS alpha activity may be related to activation levels of the default mode network (DMN), the central execution network and the salience network^35^. As we showed, responders of alpha NFT had the ability to control their brain’s self-regulation ability, which is related to remarkable alteration of the DMN^28,36^. A greater level of ECRS alpha amplitude reflects the inhibition of nonessential activity, which in turn may facilitate performance on the task^37^. In other words, a higher ECRS alpha amplitude may strongly inhibit irrelevant processes during NFT. These findings support the prediction and validation of higher learning ability during an upregulation alpha NFT regarding resting alpha activity.

A previous study showed a significant correlation between 3 learning indices and ECRS alpha activity under an NFT of increasing alpha activity^27^. Furthermore, this study extended the observation that the correlation was exclusively associated with ECRS’s alpha amplitude for 3 between-session learning indices (Table 1). Another study reported the results of predicting responders using stepwise LDA for within-session learning indices and 4 frequency bands of ECRS activity^29^. Stepwise LDA contains two major steps: one is to identify significant features, and the other is to build a model for the classification of responders. Thus, it may imply that the amplitudes of the 4 frequency bands are significant features in the previous study. However, this study found alpha activity to be an exclusive factor in stepwise LDA. The result seemed to support our observation on independence of alpha NFT success. Moreover, the accuracy using the AFB model with the L2 index was 89.1%, which is higher than the accuracy of 86.2% in a previous study^29^. These results further strengthen the independence concept of upregulation alpha NFT. Several reasons may account for these discrepancies between our results and previous findings. First, the training paradigm was different between this study and the previous study (e.g., alpha upregulation vs. alpha downregulation, long training period vs. short training period, classical alpha frequency range (8–12 Hz) vs. individual peak alpha frequency range (7.5–12.5 Hz), between-session learning index vs. within-session learning index, etc.). Second, there was a different sample size (46 of this study vs. 29 of the previous study). Third, different recording sites (central region vs. occipital region) were used. Centroparietal alpha activity plays a more important role in several cognitive functions, including attention and memory^9,11,33^.

We reported high accuracy (> 82%) in discriminating responders with 3 different learning indices (Table 2). In addition to ECRS alpha activity as a neurophysiological factor, psychological factors (including mental strategies, belief control, motivation, concentration and mood, etc.) have been investigated and reviewed elsewhere^23,24^. The prediction results of these potential psychological factors are obscure and very subjective. For a quantitative assessment, neurophysiological measures, such as ECRS alpha activity here, may be a good choice to be a determining factor for the prediction of NFT success.

The L1 index is defined as learning ability by the training parameter changes between the first session and the last session. It is most popularly used in previous studies^9,11,32,38^. Our results indicated a weak specificity of 25% for the L1 index. This raises a concern to researchers, whereas NFT responders are determined by the L1-like index.

This study demonstrated a high accuracy and sensitivity for L3 indices regarding ECRS alpha activity. However, its specificity is zero, which is unacceptable for responder classification. The L3 index calculated the temporal progression of MRAA throughout the entire training period using logistic regression analysis. It emphasized on change rate throughout the training. The underlying information of the L3 index absolutely differs from the amplitude of alpha activity. These two parameters seem to be uncorrelated or orthogonal to maximize the classification performance for responders, which may contribute to 100% vs. 0% sensitivity and specificity here. According to our results, the L3 index is suitable for responder prediction without concerns of nonresponder identification correctly.

We reported the best accuracy for the L2 index under the AFB model with good specificity and sensitivity (> 70%). Additionally, the accuracy of the current study (89.1%) was higher than the accuracy of 86.2% on NFT of downregulation alpha activity^29^ or 88.2% on NFT of upregulation beta/theta ratio^25^. The study extends our understanding of between-session validation compared with previous validation works through within-session, between-session, or mixed analysis^26,27,29^. Successful NFT learners earn experience through trial-and-error processes to control their brain activity. They often present different session-by-session variances during the training that are perhaps caused by variation of daily stress or adjustment of learning strategy and so on. The L2 index is sensitive to alteration of the entire training sessions because it considers an average MRAA across many sessions. Thus, the L2 index contained information on both alpha amplitude and temporal change across sessions, which may be a combination of the L1 and L3 indices. These specific characteristics of the L2 index may contribute to a good validation parameter using the AFB model of ECRS EEG.

In summary, we provided trainability, independence and predictability for alpha upregulation NFT. Three between-session learning indices exhibited a significant correlation with ECRS alpha activity exclusively. Moreover, the AFB model of the ECRS EEG presented better cross-validation parameters with the 3 learning indices for responder identification. This study provides a systematic analysis of NFT performance, learning indices, and cross-validation for the prediction of responders from an ECRS EEG of 2 min. Our results suggest a simple way to predict alpha training. It would be very helpful for participants and researchers to save time and may set a better route for adapting the training protocol as follows.

## Methods

The study recruited 46 healthy and NFT-naïve participants (21 females). The average age was 22.6 (SD = 1.7, range 20–27 years old). All subjects signed written informed consent prior to participation and received monetary compensation for their participation after the experiment. The study was approved by the Institutional Review Board of National Cheng Kung University Hospital, and it was conducted in accordance with the relevant guidelines and regulations.

### Experimental paradigm

Participants stayed in a quiet room and sat comfortably with our apparatus. An ECRS EEG of 2 min was recorded at the beginning. Subsequently, there were 12 NFT sessions. Each session was composed of a baseline recording of 2 min followed by six training trials of 6 min with an interval of 1 min between trials. Each participant completed 12 sessions of NFT within 4 weeks (3 days per week and a session per day).

### EEG recording and processing

EEG recording and processing of NFT in this study has been published previously^11^. Scalp voltage was recorded using a cap (Neuroscan, Inc.) embedded with six Ag/AgCl electrodes. The six electrodes formed three pairs of bipolar recordings in an anteroposterior direction (i.e., C3a-C3p, Cza-Czp, and C4a-C4p based on the international 10–10 EEG placement system), which is beneficial for characterizing frontoparietal activity, as shown in our previous topographic mapping analysis^11^. The ground electrode was at the right mastoid. Bipolar recording was used to reduce possible artifacts of head motion or eye blink^31^. All electrode impedances were ≤ 5 kΩ. The acquired signal was amplified (10,000×, 0.3–80 Hz) through a multichannel amplifier with batteries^39^, which diminished 60-Hz electromagnetic interference. These bipolar EEGs were digitized at 500 Hz (USB6009, National Instruments, TX, USA). The entire program, including acquisition and online feedback processing, was performed in the LabVIEW environment (National Instruments, TX).

Initially, ECRS EEG recording was performed for 2 min. ECRS EEG data were transformed into the frequency domain every second using a fast Fourier transform (FFT) algorithm with a Hamming window. Amplitudes within specific frequency bands, i.e., delta (1–3 Hz), theta (4–8 Hz), alpha (8–12 Hz), and beta (13–30 Hz), were calculated each second. Then, the amplitudes of these 4 frequency bands for 2 min were averaged to obtain the ECRS amplitude for each band.

In the present study, a 2-min baseline activity was recorded before each session of alpha NFT. During the baseline recording, participants freely explored the environment in front of them and were informed that eye closure was inappropriate. The baseline activity was examined to confirm less electromagnetic interference from the power line and little artifact of head or eye movement^11^. Participants were asked to avoid entering a training condition of alpha NFT during baseline recording to maintain a stable EEG quality for further analysis of MRAA.

During an NFT, EEG data were transformed into the frequency domain in a second-by-second manner using an FFT algorithm with a Hamming window. Amplitudes of 8–12 Hz from three bipolar EEGs for each 1-s epoch were averaged as feedback information, which contained an instantaneous amplitude of alpha activity on the top panel and cumulative information of available alpha amplitudes within a trial on the bottom panel^11,31^. The instantaneous alpha amplitude is indicated as a horizontal bar with a cartoon Bonny-like rabbit symbol. The length of the horizontal bar reflected the alpha amplitude of the 1-s EEG and fluctuated every second. When the Bonny index moved to the right direction, the averaged alpha amplitude was high and vice versa. Participants were instructed to move the bar to the rightmost position of the screen and to hold it there as long as possible. There was a resting period of 1 min between two consecutive trials. During each resting period, a researcher entered the room and identified timestamps of high amplitude from cumulative alpha events. Additionally, the researcher tried to understand each participant’s strategy to obtain a high alpha amplitude. Subsequently, the researcher encouraged participants to continue using the valuable strategy or provided constructive strategies according to our previous experience^11,31^. In addition, participants were informed that eye closure was not a valid strategy during the training phase. A digital camera was set in the recording room to rule out the influence of the wrong behaviors, for example, falling asleep/drowsiness, less attention during the training, or inadequate strategy involving body movement.

### Data analysis

Off-line EEG signals were analyzed to ascertain the training effect of an NFT. Spectral analysis of 1-s EEG was performed using an FFT algorithm and a Hamming window for the ECRS, baseline, and training trials. The epoch duration was 1000 ms, and the crossover percent was 0%. Then, the mean and standard deviation (SD) of 1–30 Hz amplitudes of 360 epochs in each trial were calculated. Possible artifacts were automatically marked if the EEG amplitude of a selected 1-s epoch was 2.5-fold over the amplitude SD of the trial, which worked fine to reject head or body motion artifacts^11^. Next, the processed EEGs were visualized and manually marked to remove other contaminants, such as eye blink.

The present study used the MRAA of a session as the learning performance of an NFT throughout 12 sessions. We first calculated the relative alpha amplitude through the averaged alpha amplitude of a trial divided by the averaged baseline alpha amplitude. Furthermore, the mean relative alpha amplitude (MRAA) was calculated from averaged relative alpha amplitudes across 6 trials. The data processing and analysis were performed in MATLAB (The MathWorks, Inc., Natick, MA, USA).

In the present study, we assessed learning ability from 3 different aspects of between-session factors, i.e., training parameter changes between the 1st/12th sessions, across 11 sessions on average, and across the whole training with regression^27^. For alpha upregulation NFT, participants with a positive learning index were defined as responders, and participants with a negative learning index were defined as nonresponders. The first learning index (L1) was defined as the difference in the MRAAs between the first and 12th sessions. It can be written as

1 $$\mathrm{L}1=\mathrm{MRAA}\left(12\right)-\mathrm{MRAA}\left(1\right).$$

The second learning index (L2) was calculated as the MRAA changes of the second to 12th sessions from the first session and then averaged. It can be written as

2 $$\mathrm{L}2=\frac{\sum_{i=2}^{12}MRAA(i)-MRAA(1)}{11}.$$

Moreover, we further focused on the learning speed over sessions. Considering the possible nonlinear trend of the MRAA over sessions^11^, the third learning index (L3) was the slope of the regression line calculated by a logarithmic regression model in which the session number was taken as the independent variable and the MRAA in each session was the dependent variable.

### Statistical analysis

The statistical analysis was conducted by SigmaPlot (Systat Software, San Jose, CA). A two-tailed significance level was set at P < 0.05. Data distribution was assessed by the Kolmogorov–Smirnov test. All data were found normally distributed. We examined successful training of an NFT in terms of trainability and independence. Trainability was considered a significant change in alpha amplitude across training sessions. Thus, we used one-way repeated measures analysis of variance (ANOVA) to assess the baseline alpha amplitude and MRAA across 12 sessions of an NFT. Independence was defined as a significant increase exclusively in the alpha frequency range in the present study. EEG spectra of the first and 12th sessions were compared using two-way repeated-measures ANOVA (session × frequency). If appropriate, post hoc comparisons were performed using Bonferroni correction. Pearson correlation coefficients between ECRS amplitudes of 4 frequency bands (delta, theta, alpha, and beta) and each learning index (L1, L2, L3) were compared using false discovery rate (FDR) correction by RStudio (v 1.4, http://www.rstudio.com/).

To predict responders and nonresponders, we selected a stepwise LDA^26,29^. This method contained two process stages. In the first step, the useful features were selected from all input variables by a stepwise process based on their classification effects. All input variables were the amplitudes in 4 frequency bands (i.e., delta, theta, alpha, and beta) of the ECRS EEG. Second, the coefficients of selected feature variables were determined to achieve maximum separation of two groups from the discriminant function^40^. Likewise, a discriminant function (shown as Eq. (3)) is formulated as a linear combination of the useful feature variables.

3 $$\mathrm{D}={a}_{0}+\sum_{i=1}^{n}{a}_{i}{X}_{i},$$

where *n* is the number of feature variables *X*_*i*_ and *a*_*i*_ are coefficients estimated from the input data during training to achieve maximal separation between the distributions of the discriminant scores (D) of the two groups. Consequently, we built a prediction model using the discriminant function to predict responder classification with respect to L1, L2, and L3. We assessed the performance of the prediction model using a LOOCV. The LOOCV has a smaller bias compared with other validation methods (e.g., twofold cross validation or split sample validation) in estimating the true prediction error because each observation has an equal chance of being in a training set and a test set^41^. For the prediction model, we reported its accuracy, sensitivity, and specificity, which are defined below.

4 $$\mathrm{Sensitivity}= \frac{TP}{(TP+FN)},$$

5 $$\mathrm{Specificity}= \frac{TN}{(TN+FP)},$$

6 $$\mathrm{Accuracy }= \frac{(TP+TN)}{(TP+TN+FP+FN)}.$$

True positive (TP): correctly classifying the person as responder; True negative (TN): correctly classifying the person as a nonresponder; False positive (FP): incorrectly classifying the person as responder; False negative (FN): incorrectly classifying the person as nonresponder.

## Data Availability

The datasets generated and/or analyzed during the current study will be made available upon request to the corresponding author.
